# Exploring Single-Leg Jump Testing as an Assessment of Functional Capacity in Achilles Tendinopathy: A Cross-Sectional Study

**DOI:** 10.1155/tsm2/4186587

**Published:** 2025-10-21

**Authors:** Philip Kurtz, Andrew Quarmby, Mina Khajooei, Michael Cassel, Tilman Engel

**Affiliations:** Outpatient Clinic, University of Potsdam, Potsdam, Germany

**Keywords:** Achilles, jump, single-leg, tendinopathy, testing

## Abstract

**Introduction:** This study explored single-leg jump testing as a quantitative measure of functional capacity by analyzing jump performance metrics (JPM) and the associated onset of muscle activity in Achilles tendinopathy (AT) participants compared to healthy controls (CON).

**Methods:** Twenty-four males participating in running sports (12 AT, 12 CON) performed the testing battery on a force plate, including vertical jumps, reactive jumps (RJs), and drop jumps. Analyzed JPM included ground contact time, flight time, peak force during jumps and landings, jump height, modified reactive strength index, and net impulse. Additionally, the limb-symmetry index was compared between groups along with the onset of seven lower limb muscles measured by surface electromyography.

**Results:** Multivariate comparisons showed no significant differences in JPM or limb-symmetry index between AT and CON participants. Solely, the AT group presented an earlier onset of tibialis anterior during RJs (*p*=0.005).

**Discussion:** In conclusion, single-leg jump testing in the targeted population indicated no generalized changes in overall functional capacity or onset of muscle activity, except in one jump for one specific muscle. However, the descriptively observed increased variability might suggest heterogeneous coping strategies in the presence of AT, highlighting the need for individualized assessments to detect performance deficits.

## 1. Introduction

Achilles tendinopathy (AT) often results in persistent Achilles tendon pain and loss of function related to mechanical loading [1], particularly affecting individuals involved in running sports, where its prevalence ranges from 6.2% to 9.5% [2]. Among the nine core health-related outcome domains, the International Scientific Tendinopathy Symposium Consensus recommends physical function capacity as a key quantitative measure [3, 4]. In a clinical commentary, Macdermid and Silbernagel [5] proposed that hopping and drop countermovement jump tests are useful to evaluate progress during rehabilitation, referencing a study conducted by Silbernagel et al. [6]. While this pioneering study indicated a potential for single-leg jump testing to reveal deficits in functional capacity, for example, by calculating inter-limb asymmetry, it did not evaluate the usability of multiple jump performance metrics (JPM), which have been proposed to have distinct purposes in injury rehabilitation monitoring [7], for example, following anterior cruciate ligament reconstruction [8]. Furthermore, the applicability of this assessment to an AT population experiencing mild pain while continuing to engage in running sports despite their injury remains unclear.

Additionally, several AT studies applied surface electromyography (EMG) to detect changes in the temporal course of lower limb muscle activity across tasks like hopping, walking, and running, noting alterations in the onset, offset, and duration of muscle activity [9–11]. The noted onset alterations in the studies by Smith et al. [9] and Chang and Kulig [10] suggest that AT, as a chronic condition, may manifest through altered activation patterns of lower limb muscles, potentially observable in single-leg jump testing.

Thus, the objective of this study was to explore the applicability of three different types of single-leg jumps for assessing functional capacity by analyzing JPM and the onset of muscle activity in lower limb muscles of runners with AT. It was hypothesized that (1) tendinopathic limbs present impaired JPM; (2) AT participants display greater inter-limb asymmetry across JPM; and (3) tendinopathic limbs exhibit altered muscle activation onset compared to the uninvolved limb and the dominant leg of healthy controls.

## 2. Materials and Methods

### 2.1. Participants

Twenty-four participants were sampled by convenience and enrolled in this cross-sectional study, including 12 with mid-portion AT and 12 asymptomatic controls (CON). Participants were eligible to take part given the following inclusion criteria: (1) male; (2) aged between 18 and 65 years; and (3) involved in a running sport for at least 20 km/week. Exclusion criteria were having (1) any cardiovascular, neurological, or musculoskeletal injury apart from AT; (2) surgery or illness within the past 6 months; (3) acute infection; (4) previous Achilles tendon rupture; and (5) diagnosis of insertional AT. Additionally, (6) participants with Achilles tendon pain during loading tasks > 4/10 on a Numerical Rating Scale (NRS) were excluded, aiming to differentiate long-term adaptations associated with AT from those caused by acute pain [10, 12]. Participants underwent a medical examination by a sports-orthopedic physician before being allocated to either the AT or CON group. AT diagnosis was based on the Achilles pain in history and pain on palpation during medical exam, adopting the recommended tests for clinical use from Hutchison et al. [13]. In case of a bilateral AT diagnosis, the symptomatic limb was defined by participants' self-reports regarding which side was most symptomatic during daily living and sporting activity, while the uninvolved limb was defined as the least symptomatic side, adapting the approach by Silbernagel et al. [6] and Smith et al. [9].

### 2.2. Questionnaires

Anthropometric data, including age (years), weight (kg), and height (cm), were collected from participants who then completed two self-report questionnaires: (1) the International Physical Activity Questionnaire—Short Form (IPAQ), as a comparable measure of weekly physical activity [14], and (2) the Victorian Institute of Sport Assessment Achilles questionnaire (VISA-A) [15], which measures the clinical severity of AT on a scale from 0 to 100, where 100 points indicate no Achilles tendon pain. Additionally, participants provided a subjective estimate of their weekly running distance and rated their Achilles tendon pain at rest and during physical activity on an NRS (0–10).

### 2.3. Technical Setup

After completing the initial clinical examination and self-reported questionnaires, participants were prepared for data collection. EMG was applied bilaterally to measure the onset of muscle activity using a wireless EMG capture system (band-pass filter: 5–500 Hz, gain: 5.0, overall gain: 2500, sampling frequency: 4000 Hz; Myon320, RFTD-32, myon AG, Switzerland) for recording. The muscles measured included tibialis anterior (TA), peroneus longus (PL), soleus (SOL), gastrocnemius medialis (GM), vastus medialis (VM), biceps femoris (BF), and gluteus maximus (GMAX). Bipolar EMG electrodes (2 cm inter-electrode distance, pregelled (Ag/AgCl), type P-00-S, Ambu, Mediocotest, Denmark) were positioned according to the SENIAM guidelines [16]. The skin of participants was prepared by shaving, abrasion, and cleaning with disinfectant. Skin resistance was controlled by measuring skin impedance (< 5 kΩ). Participants were provided with standardized neutral running shoes with accelerometers (ACC) attached at both heels to identify ground contact events in the EMG measurements (Myon320s, myon AG, Switzerland). A tubular bandage was applied to cover both participants' legs to reduce artifacts and movement of the equipment. A multi-axis in-ground force plate (1000 Hz, AMTI, MA, USA) was used during the jump testing battery to record ground reaction forces.

### 2.4. Protocol

Participants completed a one-minute warm-up consisting of a step-up and down exercise. During the warm-up, examiners verified signal quality of the EMG and ACC setup. The testing battery included three types of single-leg jumps: vertical jump (VJ), reactive jump (RJ), and drop jump (DJ). Participants were given the following general instructions: (1) jump as high and as quickly as possible; (2) keep hands on hips; (3) step onto the plate with the measured limb; (4) flex the inactive leg at 60°; and (5) remain still for > 2 s before jumping. Additionally, they were asked to adhere to the following jump-specific instructions. VJ: Stand upright with one leg slightly flexed, drop into a self-selected knee-bending position, and then rapidly accelerate to jump as high as possible. RJ: Start the jump identically to the VJ; upon initial landing, immediately perform another jump as quickly and as high as possible. DJ (20 cm high): Stand upright with one leg slightly flexed, flex the hip approximately 30° to lift off the standing foot, and “step off” the box without jumping. Upon initial landing, jump as quickly and as high as possible. The order of jump types and starting limb was randomized. Participants completed familiarization trials followed by three recorded trials per leg and jump type. Invalid trials were repeated.

### 2.5. Signal Processing

Force plate data were captured independently from EMG data via Vicon Nexus 2.1 (Vicon Motion Systems Ltd, UK, Oxford). Data was manually processed to isolate the Fz vector, representing the vertical ground reaction force (vGRF). A custom-built script adopted from Harry [17] and Merrigan et al. [18] operating on MatLab (Version R2023b, The MathWorks Inc., USA, Natick) was employed to extract JPM.

In the DJ and RJ, JPM were analyzed during the reactive phase of the jump, defined as follows: (1) ground contact time (GCT): time (ms) between initial ground contact and lift-off events, measured when vGRF exceeded and then dropped below 20 Newton (N); (2) flight time (FT): time (ms) between lift-off and second ground contact landing events, measured when vGRF dropped below and then exceeded 20 N; (3) peak force jump (PFJ): maximum force value (N) obtained during GCT in the reactive phase of jump; (4) peak force landing (PFL): maximum force value (N) obtained during landing after flight; (5) jump height (JH [cm]): calculated from transformed velocity data using the impulse momentum method [19, 19]; (6) modified Reactive Strength Index (mRSI): (%) calculated by *mRSI*=(*JH*/*GCT*) × 100 [20]; and (7) net impulse (NI): (*N* × *s*) calculated with the “cumtrapz” function in MatLab by subtracting body weight impulse from total impulse during the reactive phase of jump [21]. In the VJ, GCT was excluded. Additionally, inter-limb asymmetry for each JPM was expressed by the Limb Symmetry Index (LSI%) using the formula LSI%=((JPM_AT_ − JPM_CON_)/((JPM_AT_+JPM_CON_)/2)) × 100 [22].

ACC and EMG data were recorded in a synchronized way on the same software (constant latency of 16 ms, 4th order moving average filter, IMAGO record master, LabView-based, pfitec, biomedical systems, Endingen, Germany). A customized software tool was used for postprocessing of the data (IMAGO process master, LabView-based, pfitec, biomedical systems, Endingen, Germany). Data was visually inspected for artifacts, and ground contact indices were set manually using visual spikes in the ACC data. EMG data was cut and rectified, and an ensemble average of 1 s before and after ground contact was created. The ensemble average was semi-automatically analyzed for onsets of muscle activation, determined relative to ground contact when the amplitude was 3 standard deviations (SDs) greater than baseline for a minimum of 100 ms [10]. In the RJ and DJ, muscle activity onsets were analyzed during the reactive phase of the jump, while in the VJ, it was analyzed during landing after flight.

### 2.6. Statistical Analysis

Data analysis was performed using RStudio (Version 2024.4.1.748, Integrated Development Environment for R, Posit Software, PBC, Boston, MA) with additional functions included in the psych, car, stats, manova.rm, and effectsize packages. For descriptive statistics the “describeBy” function was used, reporting the mean ± SD for each group and variable. Additionally, a descriptive analysis of the onset data was conducted to identify and compare activation patterns within our population sample. The Shapiro–Wilk test and Levene test were employed to check normal distribution and variance homogeneity between variables using the “shapiro.test” and “leveneTest” functions. Global inferential tests were conducted individually for the three jump types (DJ, RJ, and VJ). Additionally, JPM derived from the force plate, the associated inter-limb asymmetry, and the EMG onset data were grouped separately for independent testing.

For comparisons between the AT and CON groups, a multivariate analysis of variance (MANOVA) was conducted using the “manova” function, with the independent variable “group” set as the between-subject factor. Comparisons within the AT group, between the symptomatic and uninvolved limbs, were conducted using the “multRM” function, with the within-subject factor set as “time.” When tests for global multivariate significance indicated significant results (*p* < 0.05), post hoc tests with Bonferroni correction were employed to calculate interaction effects. Subsequently, the “effectsize” function was used to calculate the *η*_*p*_^2^, which relates the variation explained by one factor to the variation not explained by other factors in the model [23].

## 3. Results

### 3.1. Participant Characteristics

AT participants had an average age, height, and mass of 42 ± 9 years, 182 ± 6 cm, and 79 ± 10 kg. CON participants were younger compared to the AT group, with an average of 37 ± 7 years, 181 ± 7 cm, and 77 ± 5 kg. Three out of 12 AT participants were diagnosed with bilateral AT. Both groups were classified in the “high” domain for weekly physical activity according to the IPAQ. The AT group presented with a higher overall physical activity score (4481 ± 3014 MET minutes) and average running distance (31 ± 13 km per week), compared to the CON group (3977 ± 2215 MET minutes and 28 ± 15 km). The AT group scored 73 ± 11 points on the VISA-A, indicating a lower functional status compared to the CON group (98 ± 2 points). The average duration of symptoms in the AT group was 52 ± 52 months, with tendon pain perceived as 0.6 ± 0.8 at rest and 2.9 ± 1.1 during exercise on an NRS.

### 3.2. JPM

**Figure 1:**
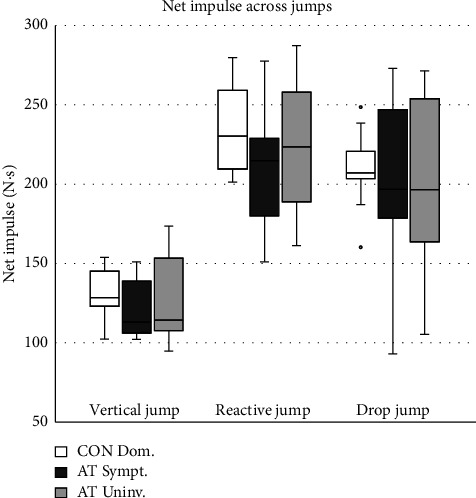
The net impulse across jumps. Box–Whisker plot (median, first and third percentiles, range, and outliers) showing the net impulse (*N *×* s*) during vertical, reactive, and drop jumps. This comparison includes the dominant leg of control participants (CON Dom.), as well as the symptomatic (AT Sympt.) and uninvolved (AT Uninv.) limbs of participants with Achilles tendinopathy.

The MANOVA indicated no significant main effects between the AT and CON groups across all JPM for the VJ (F_VJ_(7/16) = 1.2772, *p*=0.319, *η*_*p*_^2^ = 0.31), RJ (F_RJ_(7/16) = 1.2757, *p*=0.322, *η*_*p*_^2^ = 0.36), and DJ (F_DJ_(7/16) = 1.0264, *p*=0.451, *η*_*p*_^2^ = 0.31). Comparing the symptomatic limb with the uninvolved limb in the AT group using the repeated measures MANOVA also did not indicate a significant main effect within-subjects for the VJ (F_VJ_(7/16) = 1.215, *p*=0.97, *η*_*p*_^2^ = 0.12), RJ (F_RJ_(7/16) = 3.862, *p*=0.992, *η*_*p*_^2^ = 0.15), or DJ (F_DJ_(7/16) = 1.47, *p*=0.996, *η*_*p*_^2^ = 0.07). Insignificant group differences are exemplified in Figure 1, which compares the NI between limbs and shows that the AT group exhibits greater within-group variability, as indicated by longer whiskers. A comprehensive descriptive comparison, including all average JPM values between limbs, is provided in Table 1.

### 3.3. LSI%

The MANOVA did not indicate significant differences between the AT and CON groups in inter-limb asymmetry for the VJ (F_VJ_(7/16) = 1.5216, *p*=0.230, *η*_*p*_^2^ = 0.35), RJ (F_RJ_(7/16) = 1.6484, *p*=0.193, *η*_*p*_^2^ = 0.42), or DJ (F_DJ_(7/16) = 1.0624, *p*=0.430, *η*_*p*_^2^ = 0.32). See Table 2 for a detailed comparison between groups, including the average LSI(%) for each JPM.

### 3.4. EMG Onset

**Figure 2:**
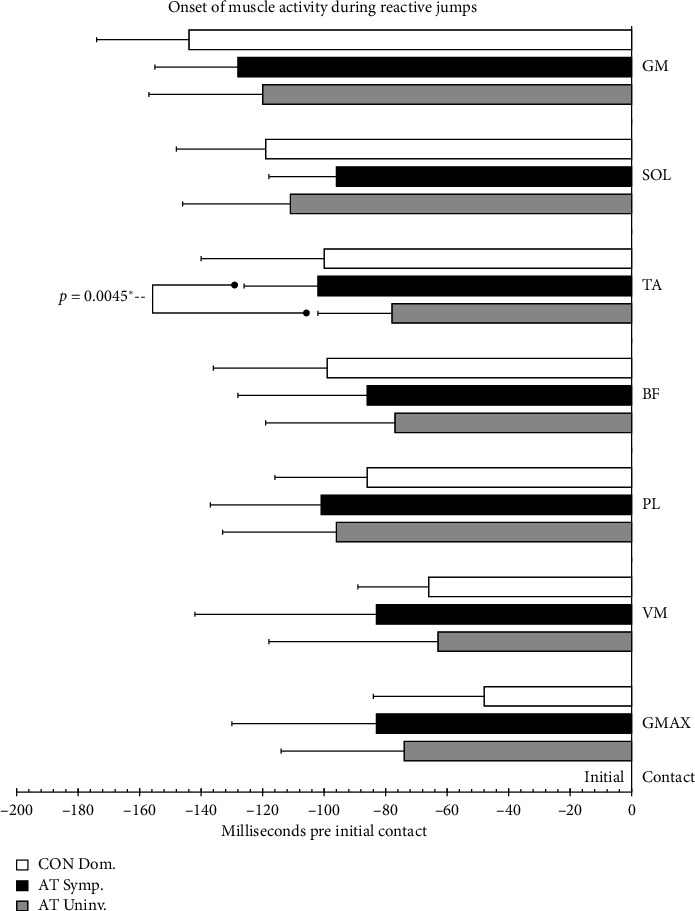
Onset of muscle activity during reactive jumps. Box plot (mean + SD) showing the onset of muscle activity in seven lower limb muscles during reactive jumps comparing the dominant leg of control participants (CON Dom.) to the symptomatic (AT Sympt.) and uninvolved (AT Uninv.) limbs of participants with Achilles tendinopathy. The muscles displayed include gastrocnemius medialis (GM), soleus (SOL), tibialis anterior (TA), biceps femoris (BF), peroneus longus (PL), vastus medialis (VM), and gluteus maximus (GMAX). An asterisk (^∗^) indicates a significant pairwise comparison using Bonferroni correction.

The MANOVA indicated no significant main effects in onset of muscle activity between the AT and CON groups for the VJ landing (F_VJ_(7/16) = 2.4985, *p*=0.0647, *η*_*p*_^2^ = 0.54), and the reactive phase of the RJ (F_RJ_(7/16) = 2.2597, *p*=0.0839, *η*_*p*_^2^ = 0.50), or DJ (F_DJ_(7/16) = 1.8503, *p*=0.1458, *η*_*p*_^2^ = 0.45). Comparing onsets between the symptomatic and uninvolved limb in the AT group using the repeated measures MANOVA achieved significance only for the RJ (F_RJ_(7/16) = 10.562, *p*=0.045, *η*_*p*_^2^ = 0.40), where *p*-adjusted pairwise comparisons revealed a significant difference for onset of TA (*p*=0.005). For a visual comparison between limbs, Figure 2 depicts the onsets of all measured lower limb muscles during the RJ. For the DJ (F_DJ_(7/16) = 5.489, *p*=0.062, *η*_*p*_^2^ = 0.38) and VJ (F_VJ_(7/16) = 1.777, *p*=0.975, *η*_*p*_^2^ = 0.07) global models did not indicate significance.  Table 3 provides a complete descriptive analysis of average onset times, including all seven limb muscles between groups.

## 4. Discussion

This study was novel in its exploration and comparison of multiple performance metrics and their associated temporal muscle activation during three different types of single-leg jumps. Results from AT participants with mild pain who continued running mostly indicated no statistically significant differences compared to a control group with similar, though not perfectly matched, age and activity levels. The main findings are (1) tendinopathic limbs did not present impaired JPM; (2) AT participants did not display greater inter-limb asymmetry; and (3) tendinopathic limbs exhibited alterations in muscle activity onset only for TA compared to the uninvolved side within the AT group during the RJ. Considering these results, none of the hypotheses can be confirmed, finding no generalizable deficits in functional capacity. However, effect sizes indicate potential for manifested functional deficits and could be meaningful for future studies involving more participants, implying that based on our data we cannot draw definitive conclusions regarding the hypotheses.

### 4.1. JPM

Of the seven tested JPM, only some descriptively indicated differences between limbs, with no proven generalizability based on the performed statistical models. Previously, Silbernagel et al. reported a significant difference of 8 ms in GCT during DJ between limbs of AT participants (*n* = 42), with even greater differences observed in the present study (12–16 ms). Despite spending more time in contact with the ground, the FT was descriptively lowest in symptomatic limbs, which also exhibited notably higher SD for both metrics, particularly in jumps with heightened reactive demand. This may hint at different adaptive strategies to maintain function, potentially reducing consistent differences between limbs. It could also explain why the SD for LSI% of JPM is elevated in the AT group, as some participants might exhibit larger inter-limb asymmetry for GCT, which could lead to smaller differences in FT, and vice versa. Descriptive statistics showed minimal differences for PFJ and PFL and their associated LSI% across groups, with both PFJ and PFL being lower in symptomatic limbs, though the differences were minor. This aligns with Azevedo et al. [24], who found no significant differences in impact forces at self-selected running speeds, and Lalumiere et al. [25], who reported no significant differences at natural and fast walking speeds. Integrating these findings, it is concluded that AT limbs do not exhibit lower peak forces, potentially reducing the usability of these metrics for detecting functional deficits. The AT group descriptively displayed lower JH, mRSI, and NI, along with increased inter-limb asymmetry for these parameters. Uninvolved AT limbs averaged 1 cm greater JH for VJ and DJ, and 5 cm for RJ, compared to the symptomatic side. These within-subject differences are consistent with Silbernagel et al. [6], who reported JH differences of 0.1 cm for VJ and 1.3 cm for DJ. The mRSI descriptively indicated effects less pronounced when comparing JH and GCT alone and was particularly sensitive to inter-limb asymmetry, which was consistently over 10% in CON and 20% in the AT group, respectively. The NI, normalized to participants' body weight and GCT, descriptively indicated the lowest values in the symptomatic limbs and the highest values in the CON group. Inter-limb asymmetry for the NI was descriptively greater in the AT group, with the biggest differences during DJ, suggesting potential impairments of AT participants to generate force efficiently. Nevertheless, despite the indication of practical applicability in five out of seven selected performance metrics, the lack of statistically significant differences between the groups limits the generalizability of the results beyond our sample and may therefore be incidental. This could be partly attributed to the targeted population, which experienced only mild pain and may have developed adaptive strategies to maintain function despite AT by sustaining high levels of physical activity.

### 4.2. Onset of Muscle Activity

Statistically only one global difference in onset of muscle activity during the RJ within the AT group was confirmed. However, descriptive analysis indicated a complex activation pattern across different jump types, with potential differences between AT and CON participants that were not substantiated by testing.

For proximal leg muscles, CON participants showed a clear sequential activation pattern during VJ and DJ: BF activated first, followed by GMAX, and then VM. In the RJ, VM activated before GMAX, deviating slightly from the pattern. This pattern was possibly altered in the AT group. Descriptively, symptomatic limbs of AT participants exhibited earlier VM and GMAX onsets, while the BF had smaller differences, and a delayed onset compared to CON participants. Descriptive differences between the uninvolved and symptomatic limbs were mostly smaller for proximal leg muscles. Interestingly, Smith et al. [9] found a delayed onset of GMAX and gluteus medius during running, possibly explained by kinematic differences between submaximal rhythmic movements like running and maximal single-leg jumps.

Three out of four distal leg muscles of CON participants showed a clear sequential activation pattern: GM activated first, followed by SOL, and then PL. This pattern was possibly altered in the AT group. Although GM was still activated first, the onset timing difference between SOL and PL was minimal. Descriptively, symptomatic limbs of AT participants exhibited earlier onsets of TA and PL, while GM and SOL were delayed compared to the CON group, with smaller within-group differences. Except for the DJ, where TA showed no delay in onset, possibly due to the different initiation of the jump by stepping off a box. The descriptively delayed activity of GM contrasts with Chang and Kulig [10], who observed earlier pre-activation of GM during submaximal hopping. However, two other studies [26, 27] indicated that primary plantar flexor activity in AT is inhibited across various movement tasks. Chang and Kulig [10] proposed that in tendinopathic limbs, synergist activity increases while antagonist activity decreases to reduce stress on the Achilles tendon. Our descriptive results indicated that both TA and PL exhibited earlier pre-activation, regardless of their opposing roles as antagonists (TA) and synergists (PL) of the main plantar flexor muscles (GM and SOL). However, the possibly earlier preactivation observed in PL might indicate an increased contribution of synergistic muscles in tendinopathic limbs, aligning with Chang and Kulig's findings.

Interestingly, variables such as jump height [28, 29], external perturbations (e.g., unstable landing surfaces) [30, 31], and internal perturbations (e.g., visual disturbances or injury) [32, 33] have been shown to modulate the onset of muscle activity. The onset alterations observed in the present study, along with the studies by Smith et al. [9] and Chang and Kulig [10], suggest that AT may act as an internal perturbation. This could potentially lead to altered activation patterns in the lower limb muscles, possibly reflecting a protective strategy. Chang and Kulig [10] theorized that in Achillis tendinosis, impaired 1b afferent input combined with a compliant tendon compromises feedback control, leading to modulation in feed-forward control of the GM, seen as earlier pre-activation during hopping. However, this study involving AT participants descriptively found the opposite effect, indicating a delayed onset in GM and SOL and earlier pre-activation of PL, VM, and GMAX. This possibly indicates a broader adaptive strategy throughout the kinetic chain to protect from further pain and injury [12, 34]. Smith et al. [9] proposed that delayed glute activity during running increases ankle joint contribution to forward propulsion, thereby enlarging the propulsive tendon load. Conversely, during single-leg jumps the descriptively earlier onsets of VM, GMAX, and PL, combined with delayed onsets in primary plantar flexors (GM and SOL), may reduce stress on the Achilles tendon. According to a prominent theory in gait mechanics, reducing the moment of one joint will increase the moment of another [35, 36]. Thus, delayed onsets of GM and SOL may reflect reduced ankle joint moment, leading to increased activity of hip and knee extensors and plantar flexor synergists, potentially indicated by earlier onsets of muscle activity. While the present study's results lack the power to confirm these hypotheses definitively, they directly support two key points from Hodges and Tucker's [12] theory of motor adaptation to pain: (1) redistribution of activity within and between muscles in an individual and task-specific manner, aimed at protecting the painful part from further pain or injury and (2) changes at multiple levels of the motor system, including motor planning adjustments, such as adopting a more protective strategy in advance of movement, like altering the sequence of muscle activation.

### 4.3. Limitations

Some limitations of this study should be recognized. The cross-sectional design limits the ability to determine whether observed alterations are causes or consequences of AT. The small sample size, due to recruitment challenges and strict inclusion criteria, led to lower statistical power mediated by reporting effect sizes to aid future researchers in designing more robust studies. Based on an average effect size of *η*_*p*_^2^ = 0.40 in outcome variables, 30 participants would have been needed instead of 24 to achieve a power of 0.80. Including participants only if their AT-associated pain was rated < 4 on an NRS is both a strength and limitation of this study, which might have influenced the results and may explain differences in outcomes compared to other studies. Only 3 out of 12 AT participants were diagnosed with bilateral AT, potentially reducing the likelihood of observing significant inter-limb asymmetry. Large SDs, particularly in the AT group, indicate high within-group variability and a broad range of performance within JPM, which reduced statistical power and challenged the assumptions of the MANOVA. These factors likely increased the probability of type II errors, affecting the study's results.

## 5. Conclusions

The present study found no overall differences in functional capacity or onset of muscle activity in the targeted population, as assessed by single-leg jumps and their associated temporal muscle activity. These findings suggest that quantifying functional deficits in runners with AT who experience only mild pain may be challenging. The increased variability observed descriptively might indicate heterogeneous coping strategies in the presence of AT, highlighting the need for individualized assessments to detect performance deficits. Descriptive analysis within our sample hinted at a possible protective strategy through the redistribution of muscle activity, although these hypotheses were not confirmed through testing.

## Figures and Tables

**Table 1:** Jump performance metrics during vertical, reactive, and drop jumps.

Group	GCT (ms)	FT (ms)	PFJ (N)	PFL (N)	JH (cm)	mRSI (%)	NI (N* *×* *s)	*p*	*η*_*p*_^2^
*Vertical Jump*									
CON Dom.	—	329 ± 34	1395 ± 149	2350 ± 269	15 ± 3	22 ± 8	131 ± 18	0.319	0.31
AT Sym.	—	295 ± 39	1481 ± 219	2406 ± 405	12 ± 4	19 ± 9	121 ± 20		
								0.970	0.12
AT Uninv.	—	317 ± 42	1467 ± 209	2558 ± 564	13 ± 4	20 ± 7	128 ± 26		

*Reactive Jump*									
CON Dom.	322 ± 51	305 ± 43	2347 ± 344	2325 ± 304	24 ± 6	151 ± 42	234 ± 27	0.322	0.36
AT Sym.	338 ± 85	267 ± 66	2367 ± 524	2246 ± 526	18 ± 6	117 ± 53	209 ± 36		
								0.992	0.15
AT Uninv.	326 ± 70	294 ± 54	2462 ± 558	2375 ± 514	21 ± 6	135 ± 47	223 ± 39		

*Drop Jump*									
CON Dom.	313 ± 48	304 ± 33	2210 ± 297	2500 ± 283	19 ± 3	123 ± 30	209 ± 24	0.451	0.31
AT Sym.	345 ± 78	287 ± 50	2290 ± 506	2485 ± 510	18 ± 7	109 ± 60	198 ± 55		
								0.996	0.07
AT Uninv.	321 ± 50	288 ± 53	2377 ± 475	2509 ± 521	18 ± 8	118 ± 59	201 ± 52		

*Note:* Mean ± SD of jump performance metrics for dominant limbs in the control group (CON Dom.) as well as the symptomatic (AT Sym.) and uninvolved limbs (AT Uninv.) in the Achilles tendinopathy group. *p*, *p* value;*η*_*p*_^2^, partial eta squared (effect size).

Abbreviations: FT, flight time; GCT, ground contact time; JH, jump height; PFJ, peak force jump; PFL, peak force landing; mRSI, modified Reactive Strength Index; NI, net impulse.

**Table 2:** Comparison of interlimb asymmetries between groups during vertical, reactive, and drop jumps.

Group	GCT	FT	PFJ	PFL	JH	mRSI	NI	*p*	*η*_*p*_^2^
*Vertical Jump*									
CON	—	5.8 ± 5	3.9 ± 4	11.9 ± 10	12.4 ± 15	13.8 ± 14	6.2 ± 5	0.230	0.35
AT	—	8.6 ± 16	6.2 ± 5	9.4 ± 6	16.6 ± 28	26.7 ± 17	8.8 ± 14		

*Reactive Jump*									
CON	5.4 ± 3	4.3 ± 4	5.3 ± 5	10.5 ± 7	8.7 ± 7	12.2 ± 7	4.4 ± 3	0.193	0.42
AT	9.2 ± 10	12.6 ± 14	7.9 ± 9	11.6 ± 12	16.8 ± 13	20 ± 15	8.3 ± 6		

*Drop Jump*									
CON	6.4 ± 5	5.8 ± 3	7 ± 3	7.3 ± 4	14.5 ± 13	19.5 ± 14	7.2 ± 7	0.430	0.32
AT	11.1 ± 4	8.3 ± 7	8.4 ± 5	7.3 ± 6	23.7 ± 22	28.6 ± 26	12.1 ± 11		

*Note:* Symmetry differences for the limbs calculated by the Limb Symmetry Index (LSI%) are presented as percentages ± SD (symptomatic/uninvolved) for the Achilles tendinopathy (AT) group and (nondominant/dominant) for the control group (CON). *p*, *p* value; *η*_*p*_^2^, partial eta squared (effect size).

Abbreviations: FT, flight time; GCT, ground contact time; JH, jump height; PFJ, peak force jump; PFL, peak force landing; mRSI, modified Reactive Strength Index; NI, net impulse.

**Table 3:** The onset of muscle activity for each muscle during vertical, reactive, and drop jumps.

Group	TA (ms)	PL (ms)	SOL (ms)	GM (ms)	VM (ms)	BF (ms)	GMAX (ms)	*p*	*η*_*p*_^2^
*Vertical Jump*									
CON Dom.	76 ± 39	88 ± 23	108 ± 33	144 ± 45	59 ± 28	95 ± 30	69 ± 24	0.0647	0.54
AT Sym.	84 ± 43	106 ± 30	109 ± 33	134 ± 34	111 ± 69	87 ± 40	114 ± 47		
								0.975	0.07
AT Uninv.	95 ± 46	112 ± 28	116 ± 34	138 ± 33	103 ± 64	76 ± 44	106 ± 36		

*Reactive Jump*									
CON Dom.	100 ± 40	86 ± 30	119 ± 35	144 ± 30	66 ± 23	99 ± 37	48 ± 36	0.0839	0.50
AT Sym.	102 ± 24	101 ± 36	96 ± 22	128 ± 27	83 ± 59	86 ± 42	83 ± 47		
								0.045^∗^	0.40
AT Uninv.	78 ± 24	96 ± 37	111 ± 29	120 ± 37	63 ± 55	77 ± 42	74 ± 40		

*Drop Jump*									
CON Dom.	112 ± 33	111 ± 28	137 ± 36	200 ± 73	49 ± 33	102 ± 38	62 ± 36	0.1458	0.45
AT Sym.	91 ± 28	114 ± 31	126 ± 47	162 ± 34	71 ± 67	80 ± 32	85 ± 56		
								0.062	0.38
AT Uninv.	108 ± 34	100 ± 39	137 ± 86	177 ± 80	53 ± 35	90 ± 35	63 ± 73		

*Note:* Mean ± SD for the onset of muscle activity in relation to ground contact for dominant limbs in the control group (CON Dom.) as well as the symptomatic (AT Sym.) and uninvolved limbs (AT Uninv.) in the Achilles tendinopathy group. TA, M. tibialis anterior; PL, M. peroneus longus; SOL, M. soleus; GM, M. gastrocnemius medialis; VM, M. vastus medialis; BF; M. biceps femoris; GMAX, M. gluteus maximus; *p*, *p* value;*η*_*p*_^2^, partial eta squared (effect size). Bold^∗^ indicates statistically significant differences with *p* < 0.05.

## Data Availability

The data that support the findings of this study are available from the corresponding author upon reasonable request.

## Nomenclature

AT: Achilles tendinopathy
VJ: Vertical jump
RJ: Reactive jump
DJ: Drop jump
JPM: Jump performance metrics
GCT: Ground contact time
FT: Flight time
PFJ: Peak force jump
PFL: Peak force landing
mRSI: Modified Reactive Strength Index
JH: Jump height
NI: Net impulse
MANOVA: Multivariate analysis of variance
IPAQ: International Physical Activity Questionnaire—Short Form
VISA-A: Victorian Institute of Sport Assessment Achilles Questionnaire
N: Newton
ACC: Accelerometers
EMG: Surface electromyography
TA: Tibialis anterior
PL: Peroneus longus
SOL: Soleus
GM: Gastrocnemius medialis
VM: Vastus medialis
BF: Biceps femoris
GMAX: Gluteus maximus
